# A Case of Corrosive Poisoning1Read at the Buffalo Academy of Medicine.

**Published:** 1897-09

**Authors:** J. C. Brown

**Affiliations:** Smethport, Pa.


### Clinical Report.

### A CASE OF CORROSIVE POISONING.[1]

### IN WHICH THE MUCOUS MEMBRANE OF ESOPHAGUS AND A PORTION OF THE STOMACH WAS VOMITED AS A COMPLETE CAST.

By J. C. BROWN, M. D., Smethport, Pa.

ON THE 27th day of April, 1897, I was called into the country about four miles and found on my arrival, about 5 P. M., a Mr. B., 32 years old, near six feet in height, strong and robust, with an anxious expression and unable to speak above a loud whisper, though he had little or no pain. His temperature was 100°; pulse, 110. Upon examining his mouth I found the mucous membrane falling in shreds from every part of the buccal cavity and pharynx, excepting about an inch of the anterior part of the tongue and about the same area of the anterior part of the roof of the mouth.

He told me that on the day before, about 10 A. M., while walking along the road he found a bottle containing what he supposed to be whisky and drank it; that he immediately experienced a burning sensation in the mouth and throat and was taken intensely sick at his stomach; that he went into a barn, where he remained until the next morning, and that he vomited and retched nearly all of the remainder of that day and all night; was unable to swallow anything after he had taken the contents of the bottle; that he came to a hotel in Smethport in the morning about 6 A. M. and went to bed, where he remained until about noon, got up and walked home, a distance of nearly two miles over a steep hill, and that he stopped on the way at springs and tried to drink, but could not swallow a drop.

Judging from the condition of the mouth I presumed that if the material which caused it had also been taken into the stomach, there must be a great deal of destruction of mucous membrane there. I suspected, however, he might be mistaken as to swallowing it, for the symptoms did not indicate that the condition extended into the stomach, therefore I felt in doubt as to prognosis.

I left a mouthwash containing tannic acid, glycerine and listerine, also an antiseptic solution to use with an atomiser. I ordered milk and white of egg, alternated with Armour's extract of beef by enema, once in four or five hours and directed that he be rubbed

1. Read at the Buffalo Academy of Medicine.

with alcohol three or four times a day. The next day I received word that he was some better and had been able to swallow a little water. On the 29th I was called and found that at the suggestion of his mother he had swallowed two teaspoonfuls of kerosene oil, which produced a great deal of nausea and vomiting, otherwise he was about the same as when last seen by me.

He continued much in this condition—the eroded portions of the mouth turning somewhat dark from the lotion, temperature ranging between 99° and 102°, pulse between 80 and 120, spitting up a great deal of purulent material, sometimes mixed with blood—until the 7th of May, when from 2 A. M. until 5.30 A. M. he had very profuse hemorrhages, vomiting nearly a pint of blood every half hour according to the nurses. I saw him at the latter hour and gave him a hypodermic of $\frac{1}{4}$ grain morphine sulphate and 1-30 grain of strychnine nitrate, which seemed to control the hemorrhage fairly well. He received another similar hypodermic about 11 A. M. Not much hemorrhage during the day. About 5 P. M. of the same day I called, but before I got into the house the patient's brother came to me, somewhat excited, saying that his brother had just vomited something very peculiar and invited me to inspect it. He brought to me what at first sight appeared to be a blood-clot, but after washing it I found it to be the mucous membrane and submucous tissue of the esophagus and stomach. It was afterward examined by Dr. H. U. Williams, pathologist at the University of Buffalo, who found it to contain also a part of the muscular coat of the esophagus. The esophageal portion was in perfect shape with the exception of a few small holes which might have been made in the effort to expel it. The stomach portion was torn somewhat in strips, due perhaps to a more pronounced effect of the corrosive agent. The whole was about sixteen inches long. It can be seen in the museum of the University of Buffalo.

After the vomiting of this membrane the hemorrhage continued very profuse for thirty-six hours and then stopped; no hemorrhage afterward. He could swallow after this, but he was not allowed to do so for three days, and began by swallowing a few teaspoonfuls of chicken soup, then milk, and milk and brandy in small quantities, gradually increasing until May 15th, when he was taking about a quart of milk in twenty-four hours, besides some toast and one or two raw eggs. During this time the enemata of milk and extract of beef were continued, which, with the exception of once or twice, were well retained.

On the 18th of May he was taken to the Buffalo General Hospital, where he was seen by Dr. Allen A. Jones, to whom I am indebted for the invitation to appear before the Academy of Medicine to show the specimen, *i. e.*, the cast, and give a brief history of the case.

[NOTE.—In discussing the case some of the members seemed to doubt its authenticity. They could hardly believe that a person could expel the structures exhibited and still be alive nearly two weeks afterward. Of course, none could see but one result and that was death in a short time.]

He was brought home next day and withstood the journey well. He continued in about the same condition until May 30th. I soon became convinced that the food which he was taking by the stomach was not being digested, and I found afterward that it was simply passed on into the intestines, where it was stored for a time and then passed per rectum in about the same condition in which he took it. His brother informed me that at one time four or five quarts passed per rectum in an undigested condition. He was allowed food by the stomach because he craved it and retained it without distress, and as death would inevitably be the result, I thought he might as well be indulged.

On the 30th of May I tried to introduce a tube into the stomach. I could pass it to within three or four inches of the stomach, but no farther. I tried again on the following day with the same result, showing that contraction had already taken place at that point. I found afterward that the whole length of the esophagus was contracted.

After my visit on the 31st of May I did not see him again until the 10th of June, but heard from him frequently. I learned from his brother that on the 1st of June he began vomiting shreds of a brownish material, looking like portions of the cast, with a very foul odor. This continued for two or three days, followed by a yellowish material still more foul-smelling than the other and which lasted for a few days. I can form no opinion as to what this material was, as I have only the nurse's description of it. At this time taking of food by the stomach had been stopped. He continued in this condition, gradually getting weaker, vomiting and retching a great deal until he died, which was on the 24th of June. An autopsy was not permitted, I am sorry to say.

The length of time which had elapsed after he had swallowed the material and his inability, feigned or otherwise, to divulge what he did with the bottle, thwarted our efforts to ascertain the nature of the corrosive.

Corrosive poisoning is not an uncommon occurrence, but it is interesting to note that life may be prolonged, as in the present instance, for an unusual period after the ingestion of a corrosive powerful enough to destroy the mucous lining of the entire esophagus and a part at least of the stomach ; also that it is possible to take such a large amount of material into the stomach without distress afterward is remarkable.

I omitted to mention that while he could perform the act of swallowing from the time he vomited the cast up to within two weeks of his death, fluids nearly always produced coughing, owing probably to the partial destruction of the epiglottis, which allowed some of the fluid to trickle into the larynx. I should have made more strenuous efforts to keep the esophagus open if I had not been satisfied that the stomach was also destroyed, at least as far as its digestive function was concerned.

Finally, it may be stated that this patient suffered a great deal with hiccough, which I found could be stopped nearly always by depressing the tongue, as in an examination of the pharynx, and holding it down twenty or thirty seconds, after which it would be sometimes an hour and sometimes several hours before another paroxysm.

